# Nobiletin Attenuates Anesthesia/Surgery-Induced Neurocognitive Decline by Preserving the Expression of Clock Genes in Mice

**DOI:** 10.3389/fnins.2022.938874

**Published:** 2022-07-07

**Authors:** Zhuonan Sun, Ning Yang, Xixi Jia, Yanan Song, Dengyang Han, Xiaoxiao Wang, Jie Sun, Zhengqian Li, Zhiyi Zuo, Xiangyang Guo

**Affiliations:** ^1^Department of Anesthesiology, Peking University Third Hospital, Beijing, China; ^2^Research Center of Clinical Epidemiology, Peking University Third Hospital, Beijing, China; ^3^Department of Anesthesiology, University of Virginia, Charlottesville, VA, United States

**Keywords:** postoperative cognitive dysfunction, nobiletin, clock gene, neuroinflammation, surgery

## Abstract

Postoperative cognitive dysfunction (POCD) is commonly observed during the postoperative period and significantly affects the prognosis of patients. Neuroinflammation plays a vital role in the pathogenesis of POCD. Despite laboratory and clinical research over the past decades, practical pharmacological strategies for the treatment and prevention of POCD are not yet available currently. Nobiletin (NOB) is a natural polymethoxylated flavone. As an enhancer of the clock protein retinoic acid receptor-related orphan receptors (RORs), NOB has been shown to attenuate inflammation and improve cognitive decline. We speculate that NOB is a candidate for the treatment and prevention of POCD. In this study, we investigated whether and how NOB affected surgery-induced neuroinflammation and POCD in adult CD1 mice. NOB pretreatment suppressed exploratory laparotomy-induced systemic inflammation and neuroinflammation in a dose-dependent manner (< 50 mg/kg), and attenuated POCD. Moreover, NOB dose-dependently reversed the decrease of brain and muscle aryl hydrocarbon receptor nuclear translocator-like protein 1 (*Bmal1*, also known as *Arntl*) and *Ror*s expression induced by exploratory laparotomy. The expression of *Bmal* was negatively correlated with tumor necrosis factor-α (TNF-α). Our results suggest that NOB attenuated POCD, possibly via preserving the expression of *Bmal* and *Ror*s and inhibiting inflammation.

## Introduction

Postoperative cognitive dysfunction (POCD) is one of the most common peri-operative complications involving a disturbance in awareness, cognitive and attention capacity, which happens in 5–50% of surgical patients (Dasgupta and Dumbrell, 2006; Mahanna-Gabrielli et al., 2019). Importantly, POCD increases postoperative mortality and the risk for mild cognitive impairment (MCI) and dementia (Witlox et al., 2010; Salluh et al., 2015; Sprung et al., 2017). POCD impairs postoperative rehabilitation and quality of life (Steinmetz et al., 2009). Therefore, it is of great social and economic value to ease the burden of POCD. The identification for the treatment and prevention of POCD has recently been a focus of peri-operative research.

Previous studies have suggested that the pathogenesis of POCD includes neuroinflammation (Subramaniyan and Terrando, 2019), circadian rhythm disorder (Jia et al., 2020), neurotransmission abnormalities (Mahanna-Gabrielli et al., 2019), and pre-operative fragile brain (Evered et al., 2016). The theory that has gained the most consensuses emphasizes the role of neuroinflammation. Proinflammatory mediators, which are triggered by peripheral surgical stress or trauma, can pass through the impaired blood-brain barrier (BBB) and cause neuroinflammation as well as neuronal dysfunction.

Circadian rhythm disorder also plays a critical role in the pathogenesis of POCD. The circadian rhythm oscillates with a ∼24 h autonomous cell period underlying intracellular transcription-translation feedback loops (TTFL). The core clock proteins brain and muscle aryl hydrocarbon receptor nuclear translocator-like protein 1 (BMAL1, also known as ARNTL) and Clock circadian regulator (CLOCK) heterodimerize to accelerate *Period* (*Per)* and *Cryptochrome circadian regulator (Cry)* transcription. The PER/CRY heterodimer inhibits *Bmal* and *Clock* transcription. These interactions form the core loop (Sato et al., 2004; Takahashi et al., 2008). The nuclear receptors receptor-related orphan receptors (RORs) and REV-ERBs compete positively and negatively on *Bmal1* promoter element RORE, and BMAL facilitates the transcription of *Rors* and *Rev-erb*s, to accelerate or suppress its transcription, which forms the second loop and confer the core loop with stability and robustness (He et al., 2016; Nohara et al., 2019).

Circadian rhythm and clock genes take part in neuroinflammation and neurocognition. For example, the clock gene *Bmal1* is required for lipopolysaccharide (LPS)-induced interleukin-6 (IL-6) expression in the microglial BV-2 cells (Nakazato et al., 2017). RORs and REV-ERBα bind the nuclear factor of kappa light polypeptide gene enhancer in B cells inhibitor, alpha *(Iκbα)* promoter RORE and affect the IκBα/ nuclear factor kappa B (NF-κB) p65 pathway in the microglia (Delerive et al., 2001; Guo et al., 2019), and *Rev-erbα* knockout eliminates temporal differences of LPS-induced endotoxin response (Gibbs et al., 2012). Surgery and anesthesia also disturb circadian rhythm leading to severer inflammation and a higher risk for POCD. In rodents, circadian disruption and sleep deprivation increase LPS or sevoflurane-induced neuroinflammation and inhibits neuronal clock gene expression in mice (Castanon-Cervantes et al., 2010; Hou et al., 2019). Currently, it is believed that circadian rhythm disturbance and neuroinflammation are not likely to be mutually exclusive, and may be overlapped to induce POCD. However, the underlying mechanism of how these factors interact with each other is largely unknown.

**FIGURE 1:**
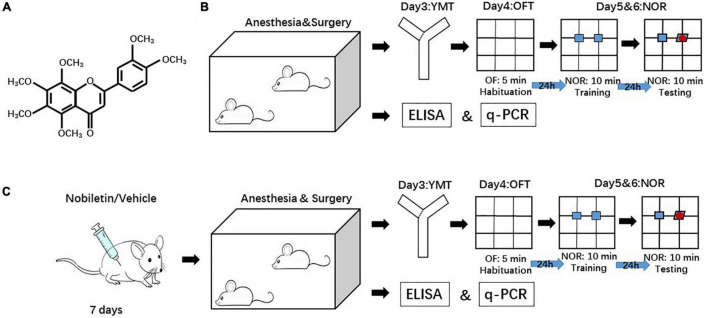
Diagram of the experimental design. **(A)** The structure of Nobiletin (NOB). **(B)** The mice received YMT at postoperative day 3, OFT at postoperative day 4 and NOR at postoperative day 5 and 6. **(C)** Another group of mice received NOB i.p. for 7 consecutive days before surgery. The behavioral tests were studies with the same interval as in panel **(B)**. YMT: Y maze test. OFT: open filed test. NOR: novel object recognition.

Despite the advance in the understanding of POCD, therapeutic strategies aiming at the prevention and treatment of POCD have not been successfully translated to the clinic. Nobiletin (5,6,7,8,3′,4′-hexamethoxyflavone, NOB) is a dietary polymethoxylated flavonoid derived from the peel and other parts of Citrus L. genus (Figure 1A). Commonly found in our diets and cuisines (e.g., orange juice, marmalade), NOB exhibits a favorable pharmacokinetic profile devoid of significant toxicity (Evans et al., 2012). Among polymethoxylated flavonoids, NOB is reported to be very effective in attenuating neuroinflammation due to a high permeability to the BBB. Its concentrations in the brain are 4 times those in the circulation (Singh et al., 2011). NOB inhibits TNF-α and IL-1β production through the IκBα / NF-κB p65 pathway in the LPS-stimulated BV-2 microglia (Cui et al., 2010). NOB protects against cell death and suppresses c-Jun N-terminal kinase (JNK) and p38 phosphorylation induced by hydrogen peroxide in the HT22 cells, a murine hippocampal neuronal model (Cho et al., 2015). Since neuroinflammation is the fundamental pathogenesis in neurodegenerative diseases and ischemia encephalopathy, NOB improves neuronal function and reduces neurocognitive decline. For example, NOB attenuates neurological deficit and cerebral ischemia injury by downregulating the expressions of NF-κB and matrix metalloprotein-9 (MMP-9) in a middle cerebral artery occlusion rodent model (Yamamoto et al., 2009; Zhang et al., 2016). NOB improves cognitive impairment induced by midazolam or constant light (Gile et al., 2018). Nobiletin improves LPS-stimulated memory impairment via MAPK and NF-κB signaling pathways (Qi et al., 2019). These results suggest that this natural compound is a theoretical candidate for the prevention and treatment of POCD. However, there is no study on NOB to test its effect on neuroinflammation and neurocognitive dysfunction after surgery.

Recent evidence has shown that NOB as a Clock-Enhancing Molecule (CEM) binds to the ligand binding domains (LBDs) of RORs to enhance their activity. Competing with REV-ERBα, RORs increases the core clock gene *Bmal1* expression, therefore, leading to multiple pharmacological actions (He et al., 2016). For example, NOB reverses the decrease of the mRNA expressions of the clock genes *Bmal1* and *Ror*s in a high-fat-diet-fed rodent model (He et al., 2016). It also attenuates the cognitive deficits induced by midazolam by preserving the clock gene *Per2* expression (Gile et al., 2018). To our knowledge, there was no research on changes in clock gene expression induced by NOB in surgical mice.

Given the clinical significance of POCD and the anti-inflammation property of NOB, we hypothesized that NOB reduced the pathogenesis of POCD. We found that NOB ameliorated POCD-like behavioral changes by decreasing systemic inflammation and neuroinflammation. These effects may be due to reversing the decrease of *Bmal1* and *Rors* gene expression induced by surgery.

## Materials and Methods

The experimental protocol was approved by the Peking University Biomedical Ethics Committee Experimental Animal Ethics Branch (Certification number: LA2020360). All animal experiments were carried out in accordance with the Basic & Clinical Pharmacology & Toxicology policy for experimental studies.

### Animal

Six- to eight-week-old male CD1 mice (weight 30 – 35 g) were purchased from the Department of Laboratory Animal Science, Peking University Health Science Centre (Beijing, China). The mice were housed in a specific-pathogen-free (SPF) room and maintained at an ambient temperature of 22 ± 0.5°C with a relative humidity of 60 ± 2% under an automatically controlled 12 h/12 h light/dark cycle (lights on at 6:00 am). Food and water were given *ad libitum*. Mice were acclimatized for 1 week prior to the experiment to enable all animals to familiarize themselves with the surroundings, staff, smells, and noises.

### Drug Administration

NOB (Solarbio, Beijing, China) was injected intraperitoneally (i.p.) daily at doses of 1, 10, 30, and 50 mg/kg body weight, respectively, in different groups at a volume of 250 μl for each dose for 7 consecutive days before surgery. NOB was prepared every day before administration. The following recipe was used to dissolve NOB: 5% dimethyl sulfoxide (DMSO) + 30% polyethylene glycol 300 (PEG300) + 10% Tween + 55% distilled H_2_O (ddH_2_O). The control-0 (C0) and the surgery-0 (S0) group received only the vehicle without NOB.

### Anesthesia and Surgery

Mice underwent exploratory laparotomy under sevoflurane anesthesia according to a previous study protocol as the model of POCD (Liu et al., 2020). Briefly, mice were exposed to 2% sevoflurane in 50% oxygen and 50% air at a flow rate of approximately 3 L/min in a transparent anesthesia chamber (RWD Life Science, Shenzhen, China), with the concentrations monitored with a gas outlet. Standard soda lime was placed in the loop to absorb carbon dioxide. The heart rate and pulse oxygen saturation of mice were monitored, and mice breathed spontaneously. After being anesthetized, mice were transferred to an electric-heating surgery table maintained at 37°C. A face mask that provided the same concentration of sevoflurane and oxygen covered the nose and mouth of the mouse to maintain anesthesia. When the depth of anesthesia met surgery requirements (no body movement with toe pinching), the exploratory laparotomy was preceded. A 1-cm ventral midline incision was performed in the abdomen, the gastrointestinal tract was exteriorized and vigorously rubbed between the surgeon’s thumb and index finger for 30 s, and the abdominal organs (liver, spleen, kidneys, and bowel) were explored gently with cotton for 25 min. The intestines were then placed back into the peritoneal cavity. The abdominal incision was closed in layers with surgical staples, and 0.1% lidocaine and EMLA cream (2.5% lidocaine and 2.5% decaine) were used for postoperative analgesia. The surgery procedure lasted for 30 min. After the surgery, mice were transferred back to the anesthesia chamber for a total of 2 h duration. Mice were taken out of the chamber and breathed in air thereafter. No response to toe pinching was observed during the whole course of anesthesia. During anesthesia and surgery, rectal temperature was monitored and maintained at 37°C. Animals tolerated sevoflurane well with all monitored variables within the physiological range. Animals in the control group were placed in their home cage with 50% oxygen and 50% air for 2 h without anesthesia, surgery, or lidocaine.

### Experimental Protocols

Three experiments were performed (Figures 1B,C). Each experiment included two or more sets of mice on different days in order to have the anesthesia start at 7:00 pm and finished at 9:00 pm. This practice was used to minimize the influence of the time difference of having surgery in affecting circadian rhythm and clock gene expression. Since the study investigated clock gene expressions, the experiment was designed to maximally simulate situations in humans, such as having surgery during an active period. Rodents are nocturnal animals. Thus, they are active during the night. The surgical time of 7 to 9 pm was chosen for it to be approved by our institute’s animal care and use committee and for the convenience of surgeons.

#### Experiment 1: The Effect of Surgery on the Inflammation and Postoperative Neurocognitive Function

To investigate the effects of surgery on the inflammatory and postoperative neurocognitive function in CD1 adult mice, mice were randomly assigned to control (C, not being exposed to surgery) or surgery (S, exposed to surgery) group (*n* = 16 each group). The levels of pro-inflammatory factors, TNF-α, IL-1β, and IL-6, in the peripheral blood, hippocampus, and prefrontal cortex 6 and 24 h after surgery were examined using enzyme-linked immunosorbent assay (ELISA) (*n* = 6 each group, randomly chosen). Three days after surgery, separate mice were started with the following behavioral tests (*n* = 10 in each group): Y maze (YM) to test cognitive ability, open field test (OFT) to study general locomotor activity and anxiety, and novel object recognition (NOR) to study recognition memory.

#### Experiment 2: Identification of the Proper Dosage of Nobiletin and Its Effects on Inflammation and Clock Gene Expression

To investigate the proper dosage of NOB, mice were randomly assigned to the following 6 groups (*n* = 6 in each group): Control-0 (C0, not being exposed to surgery, received vehicle without NOB), S0 (received surgery and vehicle without NOB), S1 (received surgery and NOB 1 mg/kg body weight), S10 (received surgery and NOB 10 mg/kg body weight), S30 (received surgery and NOB 30 mg/kg body weight), and S50 (received surgery and NOB 50 mg/kg body weight). After administration for 7 consecutive days, the mice underwent surgery the next day. These mice were sacrificed 24 h later to harvest peripheral blood and brain tissues for ELISA to detect the levels of pro-inflammatory factors, IL-1β and IL-6 (Figure 1B), and Quantitative Real-Time Polymerase Chain Reaction (qPCR) to investigate the mRNA levels of clock genes.

#### Experiment 3: The Effects of Nobiletin on Postoperative Learning and Memory Changes

After identifying the dosage of NOB at 10 mg/kg body weight, mice were randomly assigned to the following groups to investigate the effect of NOB on the postoperative neurocognitive function and its potential mechanisms (*n* = 10): Control-0 (C0, not being exposed to surgery, received vehicle without NOB), S0 (received surgery and vehicle without NOB), C10 (not being exposed to surgery, received NOB 10 mg/kg body weight), and S10 (received surgery and NOB 10 mg/kg body weight). After NOB administration for 7 consecutive days, mice underwent surgery the next day. Three days after surgery, mice were started with OFT and then NOR test.

### Blood and Brain Tissue Harvest

Mice were euthanized by deep sevoflurane anesthesia at 6 and 24 h after surgery. Blood samples were collected from the right ventricle into plastic tubes with heparin. Then mice were transcardially perfused with normal saline (NS). Their hippocampus and prefrontal cortex of both sides were dissected out and frozen to −80°C immediately for ELISA and qPCR.

### Preparation of Total Proteins From Blood and Brain Tissues

Total proteins from the blood and brain tissues were prepared. To prepare total protein extracts from the blood, blood was kept for 2 h at room temperature. The supernatant was transferred to a 2 ml epoxide vessel and centrifuged at 13,000 *g* for 20 min at 4°C. The supernatant was transferred to a 2 ml epoxide vessel and centrifuged at 13,000 rpm for 20 min at 4°C again. The supernatant was stored at −80°C for further testing.

To extract total proteins from the brain tissues, brain tissues were homogenized in RIPA buffer containing protease inhibitor cocktail and phosphatase inhibitor cocktail tablets. Homogenates were centrifuged at 13,000 rpm at 4°C for 20 min. The supernatant was transferred to a 2 ml epoxide vessel and centrifuged at 13,000 rpm at 4°C for 20 min. The supernatant was stored at −80°C for further testing. The protein concentration was determined by the BCA Protein assay kit (Thermo Scientific, United States).

### Enzyme-Linked Immunosorbent Assay

The concentration of IL-1β and IL-6 in the blood and brain was measured using ELISA kits, TNF-α (Cat. No. EMC102a, Neobioscience, China), IL-1β (Cat. No. EMC001b Neobioscience, China) and IL-6 (Cat. No. EMC004, Neobioscience, China), according to the manufacturer’s instructions. Each experimental condition was tested in two different wells and measured in duplicate.

### RNA Isolation and Quantitative Real-Time Polymerase Chain Reaction

Total mRNA was purified using the RNeasy mini kit (Cat. NO. 74014; Qiagen, Germantown, MD, United States) and treated with RNase-Free DNAase (Cat. NO. 79254; Qiagen, Germantown, MD, United States) for 30 min in room temperature. The final concentrations were measured by NanoDrop 2000 (Thermo, Waltham, MA, China). The High Capacity cDNA Reverse Transcription Kit (Cat. NO. 1725037; Bio-Rad) was used to synthesize cDNA, and SYBR green real-time PCR was performed using the Applied Biosystems QuantStudio 5 system (Applied Biosystems, CA, United States). Actin beta (*Actb*) was included as a housekeeping gene control to normalize the expression levels. The primers utilized for qPCR are presented in Table 1.

**TABLE 1:** Primers for *Rorα, Rorγ, Bmal1, Per2, Rev-erbα, Cry1* and *Actb.*

Primers	Sequences
*Rorα* Fw *Rorα* Rv	CTTCTTCCCCTACTGTTCCTTC TCTCTGCTTGTTCTGGTAGTTT
*Rorγ* Fw *Rorγ* Rv	ACAAATTGAAGTGATCCCTTGC GGAGTAGGCCACATTACACTG
*Bmal1* Fw *Bmal1* Rv	AGAGGTGCCACCAACCCATA TGAGAATTAGGTGTTTCAGTTCGTCAT
*Per2* Fw *Per2* Rv	GTCCACCTCCCTGCAGACAA TCATTAGCCTTCACCTGCTTCAC
*Rev-erbα* Fw *Rev-erbα* Rv	CGTCATCCTCTTCATCCTCCTCCTC CTTGGTAATGTTGCTTGTGCCCTTG
*Cry1* Fw *Cry1* Rv	GCCAGCAGACACCATCACATCAG GGGAAGGAACGCCATATTTCTCATCA
*Actb* Fw *Actb* Rv	GTACCACCATGTACCCAGGC AACGCAGCTCAGTAACAGTCC

### Behavioral Test

YM, OFT, and NOR were used to test POCD-like behaviors. Measures of YM, OFT, and NOR were obtained with a camera-based computer tracking system on a computer with the camera fixed to the ceiling. The enclosure was cleaned between each mouse using ethyl alcohol. Mice were taken to the experiment room 1 h before the procedure to familiarize themselves with the circumstance. The testing room had the same conditions as the housing area (e.g., lighting, temperature, etc.).

#### Y Maze

The YM is a behavioral test for short-term spatial learning in rodents. The YM apparatus has 2 equal arms and 1 different arm separated by 120°. The 2 equal arms have a dimension of 15.24 cm in length, 12.7 cm in height, and 7.62 cm in width, labeled as A and B; the one longer arm has dimensions of 20.32 cm in length, 12.7 cm in height, and 7.62 cm in width. The test consisted of 2 parts. In the first part, arm B was blocked from the opening of the arm by an opaque board with the same texture as the apparatus. The animal was placed at the end of the longer arm and allowed to explore both A and the longer arms for 5 min. In the second part, the blocking board was removed and the animal was allowed to explore the whole maze for 5 min one hour after the first part. An entry was recorded when the center of the mouse enters an arm of the maze. A number of arm entries and duration in the arms were scored.

#### Open Field Test

The OFT is a common measure of spontaneous exploratory behavior and general activity in rodents, which could reflect the anxiety and locomotor activity of rodents. The study apparatus was a 40 × 40 × 40 cm square enclosure with black plastic surrounding walls. Mice were placed in the corner nearest to the experimenter (to avoid the experimenter’s movement affecting the video recording), facing the corner. Mice were allowed to explore the enclosure for 5 min. Total distance moved (meters), frequency (number of times for mice to step into the center), and time spent in the center of the open field were recorded and analyzed.

#### Novel Object Recognition Test

The NOR tests non-hippocampus-dependent learning and recognition memory in rodents. The test consisted of 1 day of habituation, and 1 training day followed by the testing day. Mice could explore the arena for 5 min on three days. On the training day, two of the same objects were placed in the arena, 5 cm away from the walls. On the testing day, one familiar object was replaced by a novel object (approximately the same volume and height as the familiar object). Mice with the total exploration time for both objects less than 5 s in either test were excluded. The Recognition Index (RI), which is a ratio of time spent exploring the novel object relative to the total time spent exploring both objects, was calculated for indicating recognition memory.

### Statistical Analysis

Parametric results in normal distribution are presented as mean ± standard errors of the means (S.E.M). All statistical analyses were performed with GraphPad Prism 7.0 (GraphPad Software, Inc., San Diego, CA). In experiments 1 and 3, the differences between the surgery and the control group were analyzed by unpaired student *t*-test. In experiment 2, comparisons between different NOB treatment groups were assessed with one-way ANOVA followed by a Tukey’s posteriori test (Bartlett for equal variance, Brown-Forsythe for not equal variance). Linear regression analysis between proinflammatory cytokines and mRNA levels of clock genes was analyzed. In all cases, differences were considered significant at *p* < 0.05 based on two-tailed hypothesis testing.

## Results

### Nobiletin Attenuated Exploratory Laparotomy Induced Systemic Inflammation and Neuroinflammation

**FIGURE 2:**
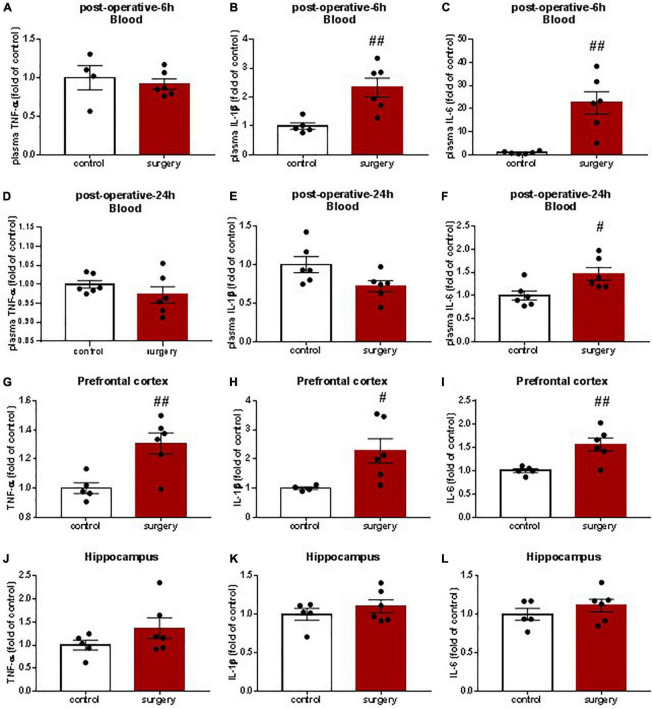
Surgery and anesthesia induced systemic and prefrontal cortical inflammation as assessed by enzyme-linked immunosorbent assay (ELISA). 6–8 week-old CD1 mice were underwent exploratory laparotomy under sevoflurane anesthesia for a total of 2 h. 6 and 24 h later blood **(A–F)**, prefrontal Cortex **(G–L)** and hippocampus **(J–L)** were harvested to examine proinflammatory cytokines TNF-α, IL-lβ and IL-6 by ELISA. The value of the control group was set to 1. ^#^*P* < 0.05, ^##^*P* < 0.01, *n* = 4∼6. Results were presented as means ± S.E.M.

To determine whether there was systemic inflammation and neuroinflammation induced by exploratory laparotomy under sevoflurane anesthesia, we tested the levels of proinflammatory cytokines TNF-α, IL-1β, and IL-6 in the blood, prefrontal cortex, and hippocampus of CD1 mice. In the surgery group, the levels of IL-1β and IL-6 at postoperative 6 h and IL-6 at postoperative 24 h in the peripheral blood were significantly increased compared with the control group but the blood levels of TNF-α at 6 h and 24 h after the surgery and IL-1β at 24 h after the surgery were not different between the two groups of animals (Figures 2A–F). The levels of TNF-α, IL-1β, and IL-6 in the prefrontal cortex at postoperative 24 h were significantly increased compared with the control group (Figures 2G–I). However, the levels of proinflammatory cytokines in the hippocampus in the surgery group were not different compared to those in the control group, though there was a tendency for higher hippocampal pro-inflammatory cytokine levels after surgery than that in the control group (Figures 2J–L).

**FIGURE 3:**
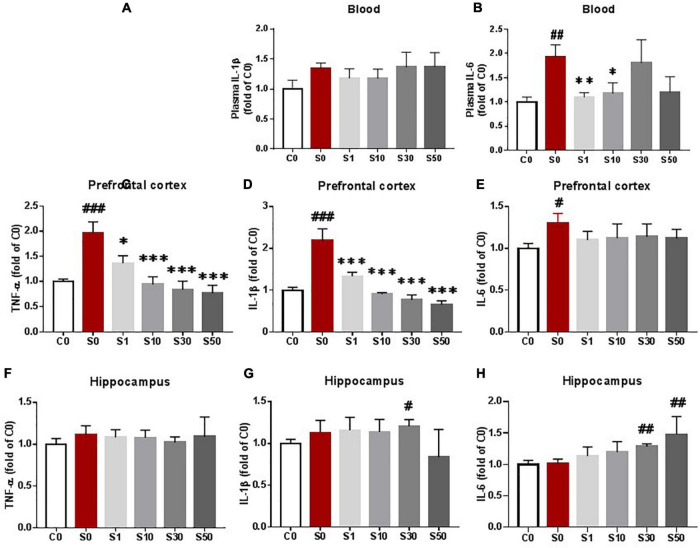
Nobiletin (NOB) attenuated systemic cytokine levels and prefrontal cortical inflammation as assessed by enzyme-linked immunosorbent assay (ELISA). Vehicle or NOB at doses of 1, 10, 30, and 50 mg/kg body weight were intraperitoneally injected, respectively, once per day for 7 consecutive days before surgery. Mice underwent exploratory laparotomy under sevoflurane anesthesia the next day after NOB pretreatment. Blood **(A,B)**, prefrontal cortex **(C,D,E)** and hippocampus **(F,G,H)** were harvested 24h after surgery to test proinflammatroy cytokine by ELISA. C0: control group without surgery; S0: surgery group with vehicle pretreatment S1,S10,S30,S50: surgery group with NOB 1,10,30, 50mg/kg pretreatment. The value of the C0 group was set to 1. # *P* < 0.05, ## *P* < 0.01, ### *P* < 0.001. *n* = 6, compared with the C0 group. *P < 0.05, **P < 0.01, ***P < 0.001, *n* = 6, compared with the S0 group. Results were presented as means ± S.E.M.

To determine an appropriate dosage of NOB to be used in subsequent studies, we tested postoperative proinflammatory cytokines in animals treated with 1, 10, 30, or 50 mg/kg body weight NOB. NOB pretreatment with 1 and 10 mg/kg decreased the level of IL-6 in the peripheral blood 24 h after surgery but not the level of IL-1β (Figures 3A,B). NOB pretreatment dramatically decreased the levels of IL-1β and TNF-α in the prefrontal cortex after surgery in a dose-dependent manner but not the level of IL-6 (Figures 3C–E). In the hippocampus, mice that underwent surgery with vehicle pretreatment showed no significant differences in cytokines compared with mice without surgery. Compared with mice who underwent surgery with vehicle pretreatment, there were no significant differences in the proinflammatory cytokines in the hippocampus of mice who underwent surgery pretreated with 1 and 10 mg/kg NOB (Figures 3F–H). However, the level of IL-1β in the hippocampus was increased at 30 mg/kg NOB compared to the control group (Figure 3G). NOB pretreatment with 30 and 50 mg/kg also caused the level of IL-6 to increase in the hippocampus (Figure 3H). Together, these findings suggest that NOB attenuates systemic inflammation and neuroinflammation in the prefrontal cortex after surgery. Given the significant anti-inflammatory effects of NOB at 10 mg/kg, we used this dosage for the following study.

### Surgery-Induced Cognitive Impairment Was Attenuated by Nobiletin

**FIGURE 4:**
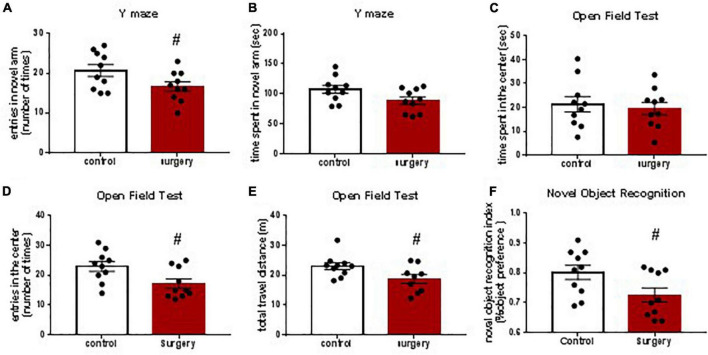
Surgery and anesthesia induced postoperative cognitive dysfunction (POCD)-like behaviors as assessed by Y maze test, open filed test and novel object recognition studies. Y maze was tested 3 days after surgery **(A,B)**. Open field test was studied 4 days after surgery **(C,D,E)**. Novel object recognition test was studied 5 and 6 days after surgery. The recognition index was calculated based on the result of the second day of NOR studies **(F)**. # *P* < 0.05, *n* = 10. Results were presented as means ± S.E.M.

To determine whether there was learning and memory dysfunction after surgery, animals were subjected to YM, OFT, and NOR tests. Our behavioral tests showed that exploratory laparotomy under sevoflurane anesthesia caused POCD (Figure 4). Specifically, the entries into the new arm of the Y maze in mice after surgery were decreased compared with that in the control group, indicating spatial memory impairment of mice after surgery (Figure 4A). There was no significant difference in the time spent in the new arm between the control and the surgery group (Figure 4B), perhaps because mice tended to stay in the start arm (the old arm) in the initial moments of the test, similar to the findings in a previous study (Kraeuter et al., 2019). In the OFT, the entries into the center and the total travel distance were decreased in mice after surgery than those in the control group (Figures 4D,E). Time spent in the center between the two groups was not different (Figure 4C). Therefore, the OFT results indicate that both locomotor ability and anxiety of mice are affected by surgery. In the NOR test, mice in the surgery group showed a reduced recognition index compared with that in the control group, validating the executive memory impairment after surgery (Figure 4F). Together, these findings suggest that surgery induces POCD-like behaviors.

**FIGURE 5:**
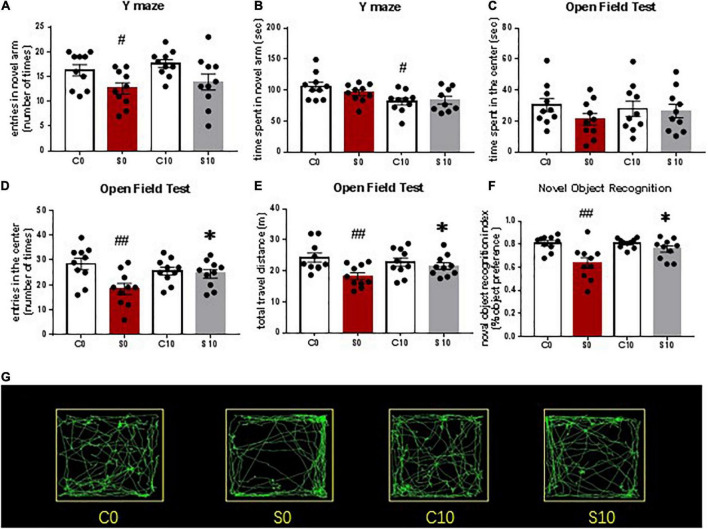
Nobiletin (NOB) 10mg/kg pretreatment mitigated postoperative cognitive dysfunction (POCD)-like behaviors after surgery and anesthesia, as assessed by Y maze test, Open filed test and novel object recognition studies. Y maze was tested 3 days after surgery **(A,B)**. Open field test was studied 4 days after surgery **(C–E)**. Novel object recognition test was studied 5 and 6 days after surgery. **(F)** The recognition index was calculated based on the result of the second day of novel object recognition (NOR) studies. **(G)** Representative OFT paths of mice in different groups. C0: control group without surgery; S0: surgery group with vehicle pretreatment; C10: control group with NOB 10mg/kg pretreatment; S10: surgery group with NOB 10mg/kg pretreatment; # *P* < 0.05, ## *P* < 0.01, *n* = 10, compared with the C0 group. *P < 0.05, *n* = 10, compared with the S0 group. Results were presented as means ± S.E.M.

To identify the role of NOB on postoperative neurocognitive function, we examined the effects of NOB pretreatment at a dose of 10 mg/kg on mice after surgery. Compared with the control group without surgery, surgery mice pretreated with vehicle developed a learning and memory impairment as described above (Figure 5). Compared with the surgery group with vehicle pretreatment, surgery mice with NOB pretreatment showed no cognitive improvement in the YM test (Figures 5A,B). After NOB pretreatment, entries into the center area and total travel distance of mice with surgery in the OFT were significantly increased, indicating an improved locomotor ability and reduced anxiety state (Figures 5D,E). NOB pretreatment also increased the recognition index of surgery mice in NOR, indicating an executive memory improvement (Figure 5F). These results suggest that NOB pretreatment attenuates postoperative cognitive impairment. Together with the previous proinflammatory cytokine data, these results suggest that NOB ameliorates inflammatory-mediated POCD-like behaviors after surgery.

### Nobiletin Attenuated Inflammation in the Prefrontal Cortex by Reversing the Decrease of *Bmal1* and *Rors* Gene Expression

We examined clock gene transcription levels to identify the mechanisms of the RORs enhancer NOB in neuroinflammation attenuation. *Rors* family consists of α, β and γ isoforms. *Rorα* and *Rorγ* are involved in cerebellum development, immune responses, and lymph node organogenesis, while *Rorβ* is exclusively expressed in the areas of the central nervous system that are involved in processing sensory information. Therefore, we chose to test the mRNA levels of *Bmal1, Rorα, Rorγ, Rev-erbα, Per2*, and *Cry1*.

**FIGURE 6:**
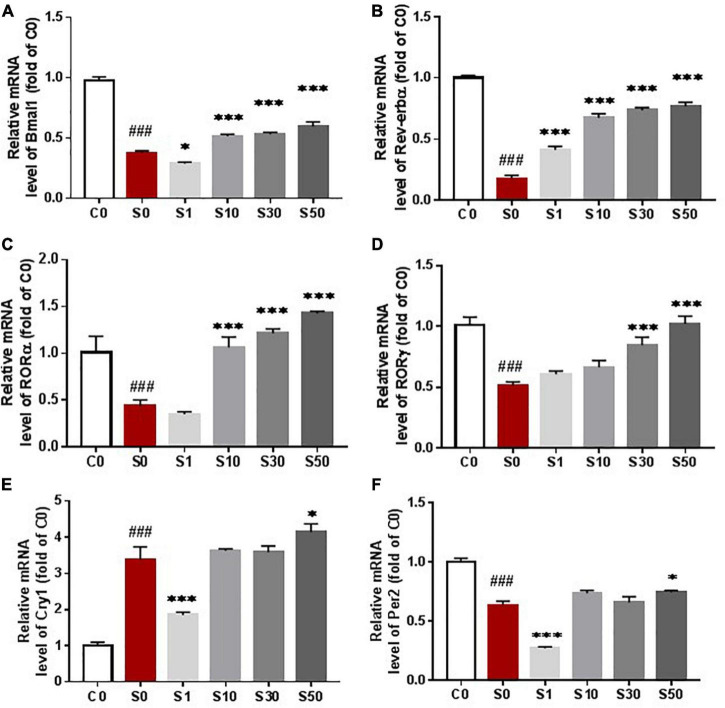
Nobiletin (NOB) reversed the decrease of clock gene expression induced by surgery under anesthesia. NOB dose-dependently reverse the decrease of Bmal mRNA induced by surgery under anesthesia at dose 10, 30 and 50 mg/kg **(A)**. NOB also reversed the mRNA decrease of Nrldl **(B)**, RORa **(C)** and RORγ **(D)** at dose of 10, 30 and 50 mg/kg **(C)**. NOB did not influence cryl **(E)** or per2 **(F)** mRNA level with dose of 10, 30 and 50mg/kg. Prefrontal cortex were collected to measure mRNA levels of Nrldl, Bmal1, RORα, RORγ, Per2 and Cryl by qRT-PCR assay. C0: control group without surgery; S0: surgery group with vehicle pretreatment; SI, S10, S30, S50: surgery group with NOB 1,10, 30, 50mg/kg pretreatment; mRNA levels were normalized to C0. # *P* < 0.05, ## *P* < 0.01, ### *P* < 0.001, *n* = 6, compared with the C0 group. *P < 0.05, **P < 0.01, ***P < 0.001, *n* = 6, compared with the S0 group. Results were presented as means ± S.E.M.

mRNA levels of *Bmal1, Rev-erbα, Rorα, Rorγ*, and *Per2* were all dramatically down-regulated in mice after surgery pretreated with vehicle compared with the control group (Figures 6A–D,F), while mRNA levels of *Cry1* were increased (Figure 6E). NOB dose-dependently reversed the decrease of *Bmal1* mRNA level, suggesting that NOB activates the RORs activity (Figure 6A). Combining with the results that NOB decreased proinflammatory cytokines in the prefrontal cortex in a dose-dependent manner, these results support that NOB attenuates neuroinflammation via a ROR-*Bmal1* pathway. The heterodimer BMAL/CLOCK accelerates *Rors* and *Rev-erbα* transcription. We found that NOB reversed the decrease of *Rors* and *Rev-erbα* induced by surgery in a dose-dependent manner (Figures 6B–D). In addition, NOB did not influence the mRNA levels of *Per2* or *Cry1* at doses of 10 and 30 mg/kg (Figures 6E,F). PER and CRY combine to form a PER-CRY heterodimer and construct another circle in the circadian self-circulation loops (Takahashi et al., 2008).

**FIGURE 7:**
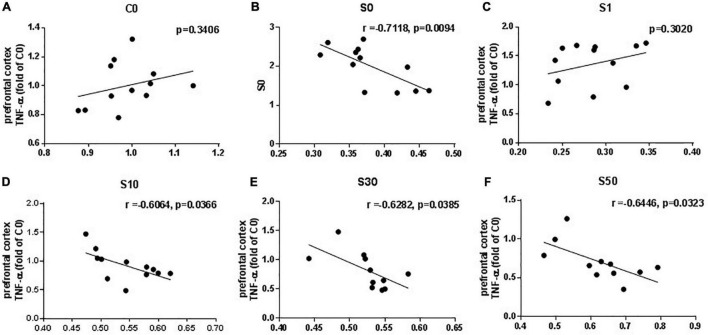
Linear regression analysis between Bmal1 mRNA level and proinflammatory cytokines TNF-α in the prefrontal cortex induced by exploratory laparotomy under sevoflurane anesthesia. The mRNA level of Bmal1 was negatively correlated with TNF-α in the prefrontal cortex in the surgery group pretreated with vehicle **(B)** and nobiletin (NOB) at doses off 10 **(D)**, 30 **(E)** and 50 mg/kg **(F)**. However, there was no linear correlations between Bmal1 mRNA level and TNF-α in the control group without surgery **(A)**, or the surgery group with NOB 1mg/kg **(C)**. C0: control group without surgery: S0: surgery group with vehicle pretreatment; S1, S10, S30, S50: surgery group with NOB 1,10,30, 50mg/kg pretreatment; *n* = 10–12.

We tested whether clock gene mRNA levels were related to the level of the proinflammatory cytokine TNF-α. The mRNA level of Bmal1 was negatively correlated with the level of TNF-α in the surgery groups with the vehicle and NOB pretreatment at 10, 30, and 50 mg/kg (Figures 7B,D–F). Combining with the previous results that NOB pretreatment elevated *Bmal1* mRNA levels and decreased proinflammatory cytokines in dose-dependent manners, these results suggest that NOB mitigates proinflammatory cytokines after surgery through the ROR-*Bmal1* pathway. However, no linear correlations between *Bmal1* mRNA level and the level of proinflammatory cytokine TNF-α were observed in the control group without surgery (Figure 7A) or surgery group with 1 mg/kg NOB (Figure 7C).

**FIGURE 8:**
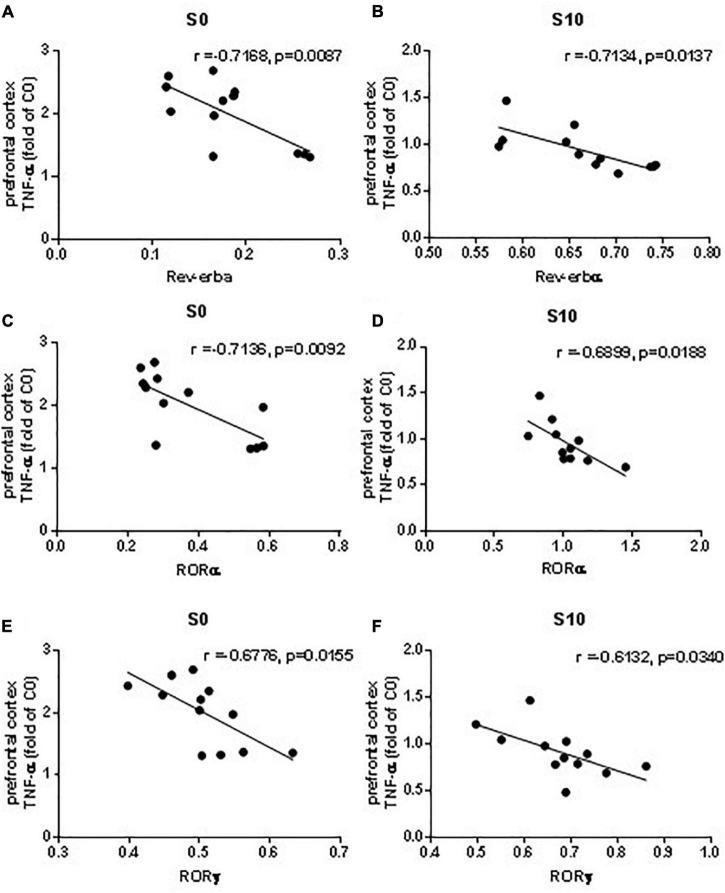
Linear regression analysis between mRNA levels of clock genes Rev-erbα, Rorα and RORγ and proinflammatory cytokine TNF-α in the prefrontal cortex induced by exploratory laparotomy. The mRNA level of Rev-erbα **(A)**, Rorα **(C)** and RORγ **(E)** in the surgery group with vehicle pretreatment correlated with TNF-α. The mRNA levels of Rev-erbα **(B)**, Rorα **(D)** and RORγ **(F)** in the surgery group with NOB 10mg/kg pretreatment correlated with TNF-α. *n* = 10–12.

The mRNA level of *Rev-erbα*, *Rorα*, and *Rorγ* in the surgery group with vehicle pretreatment was correlated with the level of TNF-α (Figures 8A,C,E). In the surgery group treated with 10 mg/kg NOB, we also observed negative linear correlations between the mRNA levels of *Rev-erbα, Rorα, and Rorγ* and the level of TNF-α (Figures 8B,D,F).

## Discussion

Our study investigated whether and how NOB affected surgery-induced neuroinflammation and POCD-like behaviors in CD1 mice. We showed that NOB pretreatment attenuated the increase of systemic and prefrontal cortical cytokine levels induced by surgery in a dose-dependent manner, and attenuated POCD-like behavior in CD1 mice. NOB also dose-dependently reversed the decrease of *Bmal1* expression and consequently reversed the decreased expression of its target genes *Rev-erbα* and *Rors* induced by surgery, indicating involvement of the ROR-*Bmal* pathway in these NOB effects.

Our study showed that the levels of proinflammatory cytokines, TNF-α, IL-1β, and IL-6, were increased in the blood and prefrontal cortex of adult CD1 mice after exploratory laparotomy under sevoflurane anesthesia for a total duration of 2 h, accompanied by POCD-like behaviors. It comes to a consensus that neuroinflammation plays a key role in POCD pathogenesis. Studies have shown that the levels of proinflammatory cytokines, TNF-α, IL-1β, and IL-6, in the hippocampus and/or prefrontal cortex are elevated after surgery and caused neuronal dysfunction (Subramaniyan and Terrando, 2019). We did not find differences in the proinflammatory cytokine levels in the hippocampus between the control and surgery mice. One possibility for this finding is that adult but not old mice were used. The levels of proinflammatory cytokines in the hippocampus and prefrontal cortex of old mice were reported to be elevated after surgery (Li et al., 2021).

Our results of the behavioral tests appear consistent with our results on the proinflammatory cytokines because studies have shown that YM results reflect hippocampus-dependent recognition memory and NOR measures non-hippocampus-dependent recognition memory (Eckenhoff et al., 2020). The behavioral results are consistent with that in previous studies, which found postoperative neurocognitive dysfunction in these behavioral tests (Peng et al., 2016; Song et al., 2018). Together, our results of proinflammatory cytokines and behavioral tests indicate neuroinflammation in the prefrontal cortex and non-hippocampus-dependent neurocognitive dysfunction after surgery and anesthesia.

Our results show that surgery under anesthesia inhibits the expression of clock genes. NOB restores the expression of *Bmal1* and its target genes *Rev-erbα* and *Rors* in dose-dependent manners. There are multiple studies on the inhibition of inhaled anesthetics or LPS on the expression of clock genes (Kadota et al., 2012; Gökmen et al., 2017; Gile et al., 2018). For example, *Bmal1, Clock, Per2*, and *Cry2* expression levels in the brain are markedly suppressed after isoflurane inhalation (Gökmen et al., 2017). However, there was also a study that showed that *Per1* expression was increased after LPS inoculation (Paladino et al., 2014). In our study, the expressions of *Bmal1, Rev-erbα, Rorα, Rorγ*, and *Per2* were decreased after surgery, while mRNA levels of *Cry1* were dramatically increased after surgery. Clock genes oscillate in a cosine curve, and surgery and anesthesia alter the rhythm of clock gene expression by changing amplitude and phase (Challet et al., 2007; Xia et al., 2015). Therefore, the pattern of changes of clock genes between intervention and control groups may alter when the assessment is made after different time intervals after the intervention. For example, the expression of clock genes is increased in the control group compared with that in the anesthesia group at certain time points (Gökmen et al., 2017). A better solution for studying clock gene expression would be continuous assessment within a 24-h period after an intervention to construct a changing curve.

**FIGURE 9:**
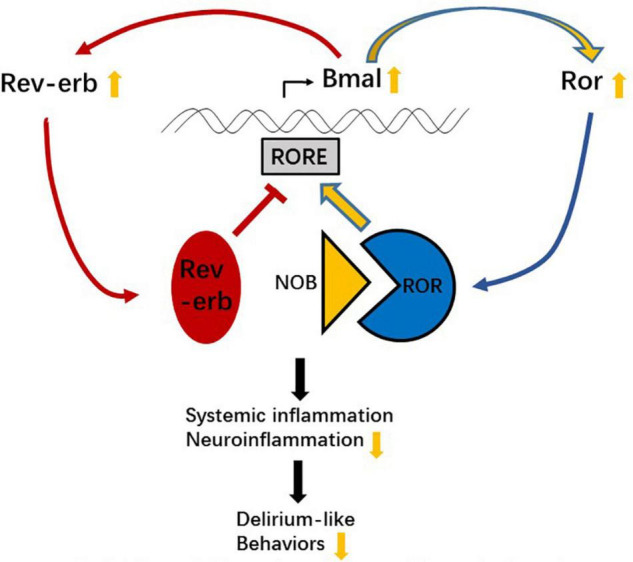
Schematic illustration of the possible mechanism of nobiletin (NOB) attenuating inflammation and postoperative cognitive dysfunction (POCD)-like behaviors.

Our result indicates that NOB functions as a ROR enhancer and boosts the mRNA levels of *Rors* through accelerating *Bmal* mRNA expression in a dose-dependent manner, which may accelerate ROR translation and form a positive feedback loop (Figure 9). Despite a dose-dependent effect of NOB on *Rev-erbα* and *Rors* expression, the expression of *Bmal1* was lower at a dose of 1 mg/kg NOB than that of the surgery group with vehicle pretreatment. Combining with the results of mRNA levels of *Rev-erbα* and *Rors* at 1 mg/kg NOB, the reduced *Bmal1* levels may be the result of the competition between non-significantly changed ROR mRNA levels and significantly increased *Rev-erbα* mRNA levels at this dose of NOB. The dose-dependent different effect of NOB on *Bmal1* expression was also reported in previous research. NOB at 10 μM was shown to decrease *Bmal1* expression, although NOB at other doses increases Bmal1 (He et al., 2016). These results indicate a complex pattern of changes caused by NOB.

The RORs and REV-ERBs bind to the RORE and are involved in multiple physiological processes, including the regulation of metabolism, immunity, and circadian rhythm. As mentioned above, there is a ROR response element (RORE) in the *Bmal1* promoter, leading to a positive and negative effect of RORs and REV-ERBs on the *Bmal1* transcription, bringing stability and robustness to circadian rhythm. Interestingly, the ROR/REV-ERB pathway has a complex effect on immune and inflammatory responses. On one hand, RORα binds to RORE in the Iκbα promoter to inhibit TNF-α-induced IL-6 level increase through the Iκbα/NF-κB p65 signaling, and staggerer mice with a deletion in the RORα gene exhibit enhanced susceptibility to inflammation (Delerive et al., 2001). On the other hand, the inverse agonist of RORγt (an isoform of RORγ, encoded by *Rorγ*) digoxin inhibits murine T helper 17 cell differentiation and reduces the severity of autoimmune disease in mice (Huh et al., 2011). A REV-ERBα agonist can also bind to the RORE of *Iκbα* and inhibit the levels of TNF-α and IL-6 through the IκBα/NF-κB p65 signaling in the BV2 cells (Guo et al., 2019). It is possible that both agonists and antagonists can fine-tune the RORs/REV-ERBs balance and exert beneficial roles. With our result, especially the unexpected mRNA level decrease of *Bmal1* at 1 mg/kg NOB, we speculate that NOB fine-tunes the RORs/REV-ERBs balance through *Bmal1* transcription, and as a result regulates *Rors* and *Rev-erbs* transcription and the transcription of their downstream target genes, like *Iκbα*, to affect inflammatory responses (Figure 9).

Examining the molecular details of NOB-ROR interaction will help to elucidate the function of ROR/REV-ERB. To our knowledge, there is no report on determining neuroprotective mechanisms of NOB via clock genes in the POCD field. Since studies have shown that NOB suppresses neuroinflammation through the Iκbα/NF-κB p65 pathway (Ihara et al., 2012; Cho et al., 2015), future research should focus on these pathways and their interaction with ROR/REV-ERB.

Linear correlations between *Bmal1* mRNA expression and proinflammatory cytokine TNF-α were not observed in the control group without surgery. Different from clock gene oscillating in cosine curves, the baseline proinflammation cytokines show circadian rhythm with biphasic patterns with multiple components, indicating other mechanisms are involved in the cytokine changes (Vgontzas et al., 2005). This suggestion may explain the non-linear correlation between the expression of clock genes and proinflammatory cytokines in the control group.

In conclusion, we have found that NOB pretreatment reduces neuroinflammation and neurocognitive dysfunction after surgery. These effects may be mediated by reversing surgery-induced suppressing effects on *Bmal1* and *Rors* expression. As a natural citrus-peel extract, NOB is a promising candidate for POCD treatment.

## Data Availability Statement

The original contributions presented in this study are included in the article/supplementary material, further inquiries can be directed to the corresponding authors.

## Ethics Statement

The animal study was reviewed and approved by Peking University Biomedical Ethics Committee Experimental Animal Ethics Branch.

## Author Contributions

ZS: initial draft writing and experiment implementing. NY and XJ: manuscript revising and methodology instructing. YS: manuscript revising. DH: design of study and methodology instructing. XW: data analysis instructing. ZL: design of the study. JS: methodology instructing. XG: acquisition of the financial support for the project leading to this publication. ZZ: design of study and manuscript revising. All authors contributed to the article and approved the submitted version.

## Conflict of Interest

The authors declare that the research was conducted in the absence of any commercial or financial relationships that could be construed as a potential conflict of interest.

## Publisher’s Note

All claims expressed in this article are solely those of the authors and do not necessarily represent those of their affiliated organizations, or those of the publisher, the editors and the reviewers. Any product that may be evaluated in this article, or claim that may be made by its manufacturer, is not guaranteed or endorsed by the publisher.
